# Juniperonic Acid Biosynthesis is Essential in *Caenorhabditis elegans* Lacking Δ6 Desaturase (*fat-3*) and Generates New ω-3 Endocannabinoids

**DOI:** 10.3390/cells9092127

**Published:** 2020-09-19

**Authors:** Sujay Guha, Serafina Calarco, M. Salomé Gachet, Jürg Gertsch

**Affiliations:** Institute of Biochemistry and Molecular Medicine, NCCR TransCure, University of Bern, Bühlstrasse 28, 3012 Bern, Switzerland; guhas@stanford.edu (S.G.); serafina.calarco@ibmm.unibe.ch (S.C.); salomegachet@gmail.com (M.S.G.)

**Keywords:** C20:4 polyunsaturated fatty acid, arachidonic acid (AA), juniperonic acid (JuA), *C. elegans*, endocannabinoids, biosynthesis

## Abstract

In eukaryotes, the C20:4 polyunsaturated fatty acid arachidonic acid (AA) plays important roles as a phospholipid component, signaling molecule and precursor of the endocannabinoid-prostanoid axis. Accordingly, the absence of AA causes detrimental effects. Here, compensatory mechanisms involved in AA deficiency in *Caenorhabditis elegans* were investigated. We show that the ω-3 C20:4 polyunsaturated fatty acid juniperonic acid (JuA) is generated in the *C. elegans fat-3(wa22)* mutant, which lacks Δ6 desaturase activity and cannot generate AA and ω-3 AA. JuA partially rescued the loss of function of AA in growth and development. Additionally, we observed that supplementation of AA and ω-3 AA modulates lifespan of *fat-3(wa22)* mutants. We described a feasible biosynthetic pathway that leads to the generation of JuA from *α*-linoleic acid (ALA) via elongases ELO-1/2 and Δ5 desaturase which is rate-limiting. Employing liquid chromatography mass spectrometry (LC-MS/MS), we identified endocannabinoid-like ethanolamine and glycerol derivatives of JuA and ω-3 AA. Like classical endocannabinoids, these lipids exhibited binding interactions with NPR-32, a G protein coupled receptor (GPCR) shown to act as endocannabinoid receptor in *C. elegans*. Our study suggests that the eicosatetraenoic acids AA, ω-3 AA and JuA share similar biological functions. This biosynthetic plasticity of eicosatetraenoic acids observed in *C. elegans* uncovers a possible biological role of JuA and associated ω-3 endocannabinoids in Δ6 desaturase deficiencies, highlighting the importance of ALA.

## 1. Introduction

Fatty acids (FAs) are endogenous and dietary constituents that have far-reaching biological roles in the animal kingdom. In eukaryotes, FAs containing two or more double bonds in the acyl chain (i.e., polyunsaturated fatty acids, PUFAs) are bioavailable and can reach cells via diet. Concurrently, indispensable enzymatic networks composed of desaturases and chain elongases generate these PUFAs (C20, C22 and C28–C34) from shorter essential FAs in situ within cells [1]. The ω-6 and ω-3 eicosatetraenoic acids (C20:4), the ω-3 eicosapentaenoic acid (C20:5) and the ω-3 docosahexaenoic acid (C22:6) constitute the main biologically important PUFAs [1]. In eukaryotes, they are both conserved and play distinct roles as constituents of membrane phospholipids, signaling molecules or substrates for oxygenases [2]. One of the most abundant and biologically relevant PUFA is the ω-6 (C20:4n6) arachidonic acid (AA), which is a major component of cellular membranes and constituent of diacylglycerols, lysophosphatidic acid and cholesteryl ester [3]. The ω-3 AA (C20:4n3) occurs in different organisms, but, rather interestingly, is the major PUFA species in *Caenorhabditis elegans* (*C. elegans*) (vide infra). In mammals, AA is oxygenated in the cyclooxygenase and lipoxygenase pathways to prostanoids and leukotrienes, respectively, among other metabolites [4]. Moreover, AA is a bona fide signaling molecule and also acts as the precursor of classical endocannabinoids (ECs), namely *N*-arachidonoyl ethanolamine (AEA, anandamide) and 1/2-arachidonoylglycerol (1/2-AG) [5]. AA occurs in algae and lower plants such as bryophytes and ferns, but is not present in angiosperms [6], which serve mainly as dietary vegetables for mammals.

Although AA can be absorbed from a carnivorous diet and is bioavailable to all tissues [7], mammals, with the exception of felines [8,9], also generate AA de novo from the essential fatty acid linoleic acid (LA) through desaturation and elongation. The rate-limiting enzyme in the biosynthesis of AA is Δ6 desaturase (FADS2), which introduces a double bond into the LA chain at C6. The Δ6 desaturase gene is expressed in all human tissues, with the highest expression in liver, heart, skin and brain [10]. The Δ6 desaturase (FADS2) knockout mouse is unable to generate PUFAs originating from both LA and α-linoleic acid (ALA) [11]. In addition to general infertility, severe dermal and intestinal ulcerations were observed, which could be abolished by supplementation of AA [12]. Furthermore, the FADS2 knockout phenotype shows severely altered membrane phospholipid compositions, as well as obesity resistance and altered lipogenesis [13]. Studies from humans suggest that Δ6 desaturase deficiencies might be associated with cutaneous abnormalities [14] and could also be related to aging [15].

The nematode *C. elegans* has become a versatile model to investigate the genetic basis of fatty acid synthesis and regulation, because most biosynthetic and metabolic pathways are conserved in this simple organism [16,17,18,19,20,21]. *C. elegans* can synthesize a broad range of PUFAs such as AA (20:4n6 and 20:4n3) and eicosapentaenoic acid (20:5n3) by using saturated fatty acid precursors obtained from *Escherichia coli* that is part of their diet. *C. elegans* shows all desaturase functions found in plants (Δ12 and Δ5 desaturase) and animals (Δ5 and Δ6 desaturase), including the n-6 and n-3 PUFA elongase functions found in animals [22]. Moreover, *C. elegans* harbors a *fat-1* gene, which encodes the enzyme n-3 PUFA desaturase that catalyzes the n-6→n-3 PUFA conversion.

Here, we employed the Δ6 desaturase mutant *C. elegans fat-3(wa22)* strain lacking Δ6 desaturase activity in order to explore the biochemical plasticity of eicosatetraenoic acid for reproduction and growth. Unlike mammals, *C. elegans* does not have any dietary fatty acid requirements and shows a dynamic conversion between ω-6 and ω-3 C18 and C20 fatty acids. Thus, *C. elegans* generates ALA from LA via Δ15 desaturase, while LA is produced via Δ12 desaturase from oleic acid [23,24]. The *fat-3(wa22)* mutant contains a serine to phenylalanine substitution at position 186 that renders the enzyme functionally inactive [22,25]. Identical to higher animals, the *C. elegans* genome encodes a single ∆6 desaturase gene (*fat-3*). The *C. elegans* ∆6 desaturase protein contains three histidine clusters distinctive of desaturases [26], showing homology to human and plant ∆6 desaturases and overall ∆6 desaturase enzymatic activity on C18 FAs [27,28].

Despite lacking ∆6 desaturase activity, the *C. elegans fat-3(wa22)* mutant is still viable when grown on C20:4 free media, notably without being able to generate AA and ω-3 AA. These animals have different growth, reproductive and neuromuscular defects that can be reversed by exogenous supplementation of AA or even EPA [25,29,30]. However, little is known about the potential compensatory biochemical mechanisms caused by the lack of AA and ω-3 AA. Moreover, the reason why this mutation is not lethal under normal growth conditions remains unknown.

In this study, the *fat-3(wa22)* mutant was characterized by targeted metabolomics focusing on eicosatetraenoic acids. Intriguingly, the ω-3 PUFA juniperonic acid (JuA), which was not known to occur in Animalia, was found together with ALA exclusively in the *C. elegans fat-3(wa22)* mutants, suggesting a biological role of this PUFA for survival. Therefore, the gross roles of JuA and ω-3 AA in worm reproduction and growth were evaluated. We compared the impact of supplementation with the different eicosatetraenoic acids on lifespan in both N2 (WT) and the *fat-3(wa22)* mutant strains. Since the biosynthesis of JuA in plants remains speculative [6], we employed specific knockdown strains to examine its potential biosynthetic pathway in *C. elegans* starting from ALA. Moreover, in the *fat-3(wa22)* mutant, we identified the ω-3 EC-like molecules 1/2-juniperoylglycerol (1/2-JG) and *N*-juniperoyl ethanolamine (JEA) derived from JuA [6] for the first time also in the animal kingdom. Additionally, in N2 (WT) animals, we measured EC-like molecules derived from ω-3 AA (20:4n3), namely ω-3 *N*-arachidonoyl ethanolamine (ω-3 AEA) and ω-3 1/2-arachidonoylglycerol (ω-3 1/2-AG) for the first time in *C. elegans*. Together with the classical ECs AEA and 2-AG, these novel ECs were tested in EC hydrolysis assays and on NPR-32 binding, a G protein coupled receptor (GPCR) shown to act as a *C. elegans* cannabinoid receptor [31]. The study provides a working hypothesis for future research on the potential compensatory mechanisms in Δ6 desaturase deficiencies in mammals.

## 2. Materials and Methods

### 2.1. Strains and Growth Conditions

The strains used for this study, wild type Bristol N2 (WT) and BX30 *fat-3(wa22)*, were cultured and maintained according to standard methods [32]. The Caenorhabditis Genetics Center (CGC) provided all the strains used in this study, including *npr-19(ok2068)* and *npr-32(ok2541).* All animals were maintained on nematode growth media (NGM) with plates seeded with *Escherichia coli* OP50 and incubated at 23 °C. For the LC-MS/MS measurement experiments, the well fed N2 (WT) and *fat-3*(*wa22*) animals were maintained on 60 mm NGM plates. Animals were collected from these plates by washing them with S-basal buffer, the worm pellets collected were washed four–five times to get rid of bacterial residue and then stored in −80 °C until the measurements were carried out. The *E. coli* and growth media did not contain eicosatetraenoic acids as analyzed by LC-MS/MS (QTRAP 4000, AB Sciex LLC, Framingham, MA, USA).

### 2.2. Dietary Lipid Supplementation

Ten mM stock solutions of AA, ω-3 AA and JuA were prepared in DMSO and kept at −20 °C. These stocks were diluted in *E. coli* OP50 to a final concentration of 200 μM, and 100 μL of the volume was seeded onto each plate, with appropriate DMSO controls, allowing drying for 1 h in the dark. The animals that were treated with the PUFAs from hatching were allowed to grow on these plates until they reached L4 larval stage.

### 2.3. Brood Size Assay and Development Monitoring

N2 (WT) and *fat-3(wa22)* were transferred onto NGM plates supplemented with PUFAs as described above. Once they reached L4 larval stage, one worm per plate was transferred to a plate freshly seeded with PUFAs and allowed to grow at 23 °C. These animals were transferred to new plates every 24 h until they ceased to lay eggs. The number of hatched progeny was checked and counts were performed after the population reached L3/L4 stage. This assay was carried out in three independent trials. Results were analyzed with GraphPad Prism 5^®^, San Diego, CA, USA. The data were pooled and analyzed using Student’s *t* test. *p* < 0.05 was accepted as statistically significant.

For monitoring the development N2 (WT) and *fat-3(wa22)* gravid animals were allowed to lay eggs for 2–3 h. These eggs were transferred onto NGM plates supplemented with PUFAs as described above. Their growth was monitored and then imaged using an AmScope MU1000 camera, USA.

### 2.4. Lifespan Assays

All lifespan assays were carried out at 23 °C. Synchronized populations were obtained by allowing 10–15 hermaphrodites lay eggs overnight, and the next day the parents were removed. The eggs were allowed to hatch and 30 L4/young adult animals per plate (NGM plate containing 50 μg/mL FUDR to prevent the growth of progeny) were used for each assay. All the assays were carried out at least in triplicates and a minimum of two independent trials were performed for all conditions. The dead animals were counted starting the next day and exploding, protruding, bagging or contaminated animals were censored if applicable. We defined the day when we transferred the L4/young adult animals as day 0 of adult age. All statistical analyses were carried out using results that were analyzed with GraphPad Prism 5^®^. Kaplan–Meier lifespan analysis was carried out, and *p* values were calculated using the log-rank test. *p* < 0.05 was accepted as statistically significant.

### 2.5. RNA Interference by Feeding

The RNA interference (RNAi) by feeding assay was performed on NGM agar plates supplemented with additional antibiotics (50 mg/mL ampicillin, 12.5 mg/mL tetracycline) and 0.8 mM isopropyl-β-D-thiogalactopyranosid (IPTG). The Ahringer RNAi collection was used, *E. coli* HT115 bacteria containing RNAi vectors expressing double-stranded RNA of the genes *fat-4*, *elo-1*, and *elo-2* were pre-cultured, induced with IPTG, and used as nematode food as described previously [33]. Assays were carried out on plates seeded with RNAi bacteria induced overnight; the PUFAs were dissolved in sterile distilled water, and then overlaid on the plates just before the assays.

### 2.6. Synthesis of the Endocannabinoid-Like Molecules Juniperoyl Ethanolamide (JEA) and ω-3 Arachidonoyl Ethanolamine (ω-3 AEA)

Synthesis of ω-3 AEA was performed as described previously [6], where we prepared JEA. In the case of ω-3 AEA, the starting material for the reaction was ω-3 AA instead of JuA. The desired compound ω-3 AEA was obtained as a colorless dense oil (2.1 mg, yield: 88%, purity > 96%). More details are shown in the supplemental information (Figure S1).

### 2.7. Synthesis of the Endocannabinoid-Like Molecules 1/2-Juniperoylglycerol (1/2-JG) and ω-3 1/2-Arachidonoylglycerol (ω-3 1/2-AG)

Synthesis of compounds ω-3 1/2-AG was performed as described previously [6], where we prepared 1/2-JG. In the case of ω-3 1/2-AG, the starting material for the reaction was ω-3 AA instead of JuA. A mixture of the two isomers 1 and 2 was obtained in a ratio 1:1 (1.8 mg, yield: 48 %). More details are shown in the supplementary materials (Figure S2).

### 2.8. Chromatographic Conditions Used for LC-MS/MS Targeted Metabolomics Analysis (Identification and Qquantification)

An API 4000 QTrap mass spectrometer equipped with a TurboIonSpray probe (AB Sciex, Framingham, MA, USA) connected to a Shimadzu UFLC was used for the quantification and identification targeted metabolites following the methodology recently published by us [6]. Only for the identification of the structural isomers of AEA (i.e., JEA and ω-3 AEA) and 1/2-AG (i.e., 1/2-JG and ω-3 1/2-AG), the chromatographic conditions were improved.

Chromatographic conditions were used for identification by LC-MS/MS of structural isomers analyzed in positive mode (i.e., AEA, JEA, ω-3 AEA and 1/2-AG, 1/2-JG, ω-3 1/2-AG).

Analytical LC separations were performed using two consecutive columns (i.e., ReproSil Saphir 100 C18 column and Reprosil-PUR C18 column) with a flow rate of 0.35 mL/min and an oven temperature of 40 °C using a gradient of CH_3_OH containing 2 mM ammonium acetate (eluent B) and water containing 2 mM ammonium acetate and 0.1% formic acid (eluent A). The gradient was as follows: 60–80% eluent B from 0–22.0 min; held at 80% B from 22.0–30.0 min, 80–99% eluent B from 30.0–31.0 min, held at 99% from 31.0–35.0 min. From 35.0–36.0 min, the column was re-equilibrated to 60% B and conditioned from 36.0–45.0 min at 60% B. The autosampler was cooled at 4 °C.

### 2.9. Quantitative Analysis of Targeted Analytes Using LC-MS/MS

Quantification was performed using external calibration standards as recently published [6] with a few modifications. The concentrations of the labeled IS used for the analysis were: 10 ng/mL for AEA-*d_4_*, 25 ng/mL for 1/2-AG-*d_5_*; 500 ng/mL for ALA-*d_14_* and 1500 ng/mL for AA-*d_8_*. The concentrations of the calibrators were 16.4–3200 ng/mL for AA; 250–10,000 ng/mL for ALA; 640–10,000 ng/mL for DHGLA; 0.5–20 ng/mL for AEA and 1.6–320 ng/mL for 1/2-AG. In order to obtain the concentration reported (nmol (or pmol)/mg protein), the analytical amount (ng/mL) was multiplied by 0.1 (to correct the volume of 1 mL to the final volume of 0.1 mL) and then normalized to the protein concentration. Calibration curves (five calibration points, run in triplicate) and control samples (*n* = 4) were run during the analysis of the samples and were used to evaluate the method’s performance. The LOD (and LOQ) were 16.4 (16.4), 100 (250), 100 (640), 0.03 (0.5) and 1.6 (1.6) ng/mL for AA, ALA, DHGLA, AEA and 1/2-AG, respectively. All analytes show linear response within the dynamic range investigated with R^2^ values of 0.997 ± 0.003, 0.995 ± 0.006, 0.991 ± 0.013, 0.997 ± 0.009 and 0.998 ± 0.003; slopes of 0.0068 ± 0.0009, 0.0048 ± 0.0008, 0.0020 ± 0.0006, 0.0526 ± 0.0072 and 0.0480 ± 0.0042; CV% of the slopes of 14, 17, 29, 14 and 9; and intercepts of −0.204 ± 0.273, −0.226 ± 0.781, 0.413 ± 0.143, 0.015 ± 0.163 and −0.103 ± 0.182; for AA, ALA, DHGLA, AEA and 1/2-AG, respectively. The accuracy is within 76–111% for AA (at concentrations of 40, 260 and 1600 ng/mL), 70–114% for ALA (at 260, 1600 and 10,000 ng/mL), 75–102% for DHGLA (at 1600 and 10,000 ng/mL), 92–129% for AEA (at 0.5, 3.2 and 20 ng/mL) and 92–129% for 1/2-AG (at 4.1, 25.6 and 160 ng/mL), while the method precision (CV%) is within 5–17% for AA, 8–19% for ALA, 8–14% for DHGLA, 7–17% for AEA and 9–12% for 1/2-AG. The recoveries evaluated upon spiking before and after extraction, at the concentrations stated above, were between 92–99% for AA, 94–110% for ALA, 96–109% for DHGLA, 96–106% for AEA and 90–103% for 1/2-AG. The quantification in wild type animals will be presented as sum of the structural isomers in N2 (WT) animals (i.e., AA + ω-3 AA, AEA + ω-3 AEA and 1/2-AG + ω-3 1/2-AG) and as juniperoyl derivatives (i.e., JuA, JEA and 1/2-JG) in *fat-3*(*wa22*), RNAi treated animals.

### 2.10. Sample Preparation for LC-MS/MS Analysis

400 µg protein was added into a 2 mL Eppendorf tube containing 800 µL CHCl3, 400 µL CH_3_OH, and 10 µL of IS solution. The worm protein homogenate was suspended in S-basal, and often needed to be adjusted to a final volume of 200 µL with PBS to yield a 4:2:1, *v*/*v*/*v* ratio (CHCl_3_:CH_3_OH:H_2_O). The mixture was thoroughly vortexed and sonicated (5 min at 4 °C) for lipid extraction. Subsequently, the sample was centrifuged at 16,100× *g* at 4 °C for 5 min. The supernatant was transferred into 1.5 mL Eppendorf tubes and evaporated to dryness using a SpeedVac Concentrator from Thermo Fischer Scientific, Switzerland. The samples were reconstituted in 100 µL ACN (final volume) and centrifuged for 5 min at 16,100× *g* at 4 °C. Eighty µL of these samples were carefully pipetted out, placed into conic amber vials and analyzed by LC-MS/MS (10 µL injection volume). Each sample was injected four times, once for quantification and once for identification of structural isomers in both positive and negative modes.

### 2.11. Biochemical Assays

Materials: all the compounds tested were solubilized in DMSO or ethanol. All the non-radiolabeled compounds (AEA, 2-AG, MAFP, JZL-195) were purchased from Cayman Chemicals (Ann Arbor, MI, USA) or Sigma Aldrich (Saint Louis, MO, USA). [Ethanolamine-1-^3^H] (60 Ci/mmol) and [1,2,3-^3^H]-2-mono oleoyl-rac-glycerol ([^3^H] 2-OG) (60 Ci/mmol) were obtained from American Radiolabeled Chemicals Inc. (Saint Louis, MO, USA), while [Side Chain-2,3,4-^3^H(N)]-CP55,940 was purchased by Perkin Elmer Life Sciences (Waltham, MA, USA). Scintillation liquid and Tri-Carb 2100 TR scintillation counter were obtained from PerkinElmer Life Science (Waltham, MA, USA). Other substances, such as PBS (Phosphate Buffer Saline) and BSA (Bovine Serum Albumin) were purchased from Sigma-Aldrich (Saint Louis, MO, USA). Worm homogenate and pig brain homogenate were prepared in our laboratory.

Worm homogenization and membrane preparation: the animals were collected from NGM plates as described before. The animals were freshly broken/homogenized by tip sonication before the beginning of every assays in S-basal and then a protein estimation was carried out for these samples, by a standard BCA kit (Sigma-Aldrich, St. Louis, MO, USA). For membrane preparations the worm pellet was resuspended in homogenization buffer (50 mM Tris HCl pH = 7.4, 3 mM MgCl_2_, 1 mM EGTA). Next, the homogenate was transferred to an ultracentrifuge tube and spun at 100,000× *g* for 60 min at 4 °C. The supernatant was carefully removed and the pellet was gently washed. Then, the pellet was resuspended with the help of a polytron in 50 mM Tris HCl pH = 7.4 and passed through a clean syringe. Finally, the sample was sonicated in a water bath for 5 min. A protein estimation for the membranes was carried out and the aliquots were frozen and stored at −80 °C.

Enzymatic hydrolysis assay of novel ECs: the enzymatic hydrolysis of the ethanolamine derivatives JEA and ω-3 AEA and the glycerol derivatives 1/2-JG and ω-3 1/2-AG was evaluated in competitive hydrolysis assays using [^3^H] AEA and [^3^H] 2-OG, respectively. The reaction was performed using worm homogenate (200 or 100 µg per sample) or pig brain homogenate (200 µg per sample) in 194 μL of assay buffer (10 mM Tris-HCl, 1 mM EDTA, 0.1 % BSA, pH 8.0). Different concentrations of JEA and ω-3 AEA were co-incubated for 20 min at 37 °C with a mixture of 1 nM [^3^H]AEA and unlabeled AEA (final 2µM), while 1/2-JG and ω-3 1/2-AG were co-incubated with a mixture of 1nM [^3^H]2-OG and unlabeled 2-OG (final 100 nM). The reaction was stopped by adding 400 μL of ice-cold mixture CHCl_3_-CH_3_OH (1:1 *v*/*v*), and, subsequently, tubes were vortexed and centrifuged for 10 min at 4 °C and 10,000 rpm. Finally, the upper aqueous phase was transferred to scintillation vials and mixed with 3 mL of Ultima Gold scintillation liquid. Signal was detected with TRICARB 2100TR scintillation counter and results were analyzed with GraphPad Prism 5^®^ and expressed as [^3^H] AEA or [^3^H] 2-OG hydrolysis (% of vehicle).

### 2.12. NPR-32 Binding Assay

The binding activities of the *N*-acylethanolamines AEA, JEA and ω-3 AEA and the glycerol esters 2-AG, 1/2-JG and *ω*-3 1/2-AG were evaluated in a competitive binding assay using the known nonselective cannabinoid CB1/CB2 receptor radioligand [^3^H]CP55,940 on membrane preparations (see biochemical assays) of N2 (WT) and *npr-32(ok2541)* mutants, respectively. Briefly, 75 μg of crude membrane preparations were resuspended in 300 μL of assay buffer (50 mM Tris-HCl, 2.5 mM EDTA, 5 mM MgCl_2_, 0.5% BSA, pH 7.5) in silanized glass tubes and preincubated with 5 µM JZL-195 for 30 min at 30 °C. Afterwards, different concentrations of AEA, JEA, ω-3 AEA, 2-AG, 1/2-JG and ω-3 1/2-AG were co-incubated with [^3^H] CP55,940 for 75 min at 30 °C. After the incubation time, membrane suspensions were rapidly filtered through a 0.1% polyethyleneimine presoaked 96-well microplate bonded with GF/B glass fiber filters (UniFilter-96 GF/B, PerkinElmer Life Sciences) under vacuum and washed 12 times with 120 µL of washing buffer (50 mM Tris-HCl, 2.5 mM EDTA, 5 mM MgCl_2_, 0.5% BSA, pH 7.5). Filters were added with 50 µL of MicroScint20 scintillation liquid, and radioactivity was measured with the 1450 MicroBeta Trilux top counter (PerkinElmer Life Sciences). The results were analyzed with GraphPad Prism 5^®^ and expressed as % of [^3^H]CP55,940 binding. IC_50_ values of each compound were estimated from fitted nonlinear curves and were used to calculate the *K*_i_ values by applying the Cheng-Prusoff equation. Specific binding was determined using the *npr-32*(*ok2541*) mutant strain in which saturation binding was abolished. Importantly, normalizing against the NPR-19 null mutant strain- *npr-19*(*ok2068*), no saturation binding curve could be achieved. In the N2 WT strain a total 30–35% of displacement was observed for the highest concentration (100 µM) of EC and EC-like compounds and data was normalized accordingly.

## 3. Results

### 3.1. Differential Expression of Eicosatetraenoic Acids in Wild Type and fat-3(wa22) Mutant C. elegans

To characterize the absence of C20:4 PUFAs we measured the major eicosatetraenoic acids in the *C. elegans fat-3(wa22)* mutant by LC-MS/MS. In agreement with a previous study [4], the strain lacks AA, ω-3 AA, but also sciadonic acid (20:3n6) (ScA), which we hypothesized to be present in this strain (vide infra) (Table 1).

**Figure 1:**
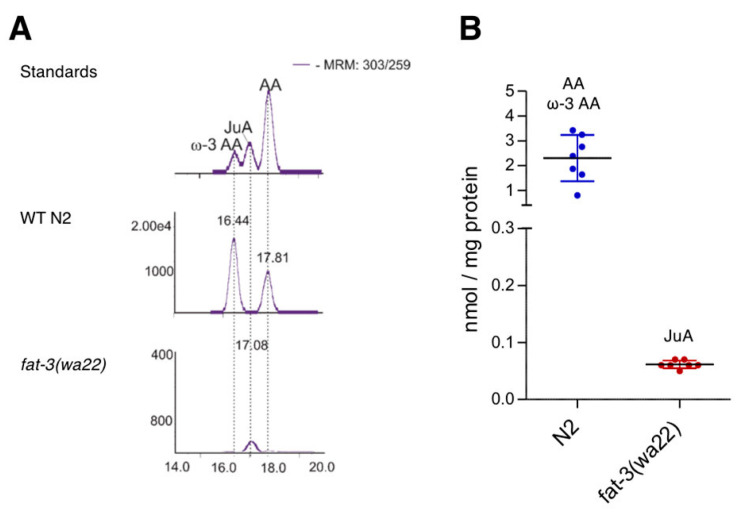
Detection and quantification of FAs in N2 (WT) and *fat-3(wa22) C. elegans*. (**A**) Chromatographic resolution in LC-MS/MS showing the presence of C20:4 FAs AA, ω-3 AA in N2 (WT) animals and new C20 FA JuA in *fat-3(wa22)* mutants. (**B**) Quantification of AA, ω-3 AA and JuA in wild type N2 and *fat-3(wa22*) mutants deficient in Δ6 desaturase activity. Scatter plot shows mean ± SD.

Unexpectedly, we detected juniperonic acid (JuA) (20:4n3) in *fat-3(wa22)* mutants and for the first time unambiguously determined this lipid in Animalia. While N2 (WT) animals showed very high amounts of ω-3 AA and AA, which in the literature are often not distinguished analytically from each other, the *fat-3(wa22)* mutant exclusively showed the presence of JuA (Figure 1A). Since JuA has an identical mass fragmentation pattern as ω-3 AA and AA but a different chromatographic behavior on a Reprosil C18 column, we were able to distinguish them. The LC-MS/MS method employed was further validated with a commercial JuA standard and quantification was carried out using deuterated AA. With approximately 0.06 nmol/mg protein JuA was present at µM concentrations in total worm homogenate (Figure 1B). As shown in Table 1, γ-linoleic acid (GLA) and dihomo-γ-linoleic acid (DHGLA) were only present in the *C. elegans* wild type N2 strain.

### 3.2. Recovery of fat-3(wa22) Mutant Related Defects by Supplementation of JuA

**Figure 2:**
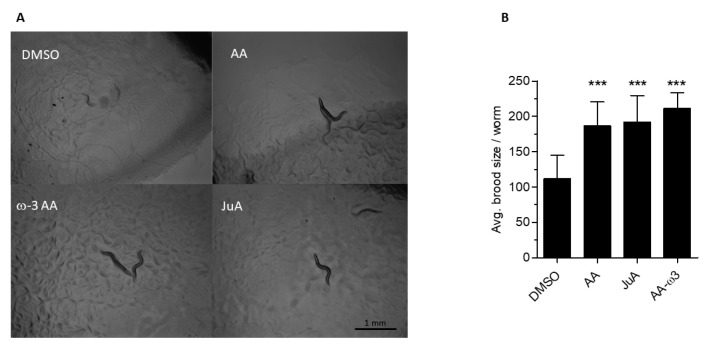
Recovery of growth defects with addition of FAs. (**A**) Recovery of growth delay after 36 h of hatching at 23 °C of *fat-3*(*wa22)* mutant with the supplementation of C20:4 FAs (Scale bar = 100 mm). (**B**) Brood size of *fat-3*(*wa22*) *C. elegans* after addition of different C20:4 FAs. Brood size experiments were carried out with *n* = 5 animals per condition in at least three independent trials (*** *p* < 0.0001). Data show mean values ± SD.

Given the unexpected presence of JuA, we hypothesized that this PUFA is generated de novo in the *fat-3(wa22)* mutant strain and might functionally compensate for the general lack of ω-3 AA and AA. To address this, JuA was supplemented to the culture media of the *fat-3(wa22)* mutants and the phenotypic changes were assessed 36 h after hatching. As reported previously [22], the mutant animals were significantly smaller (reduced thickness and length) than N2 (WT) animals, indicative of various metabolic deficiencies. Supplementation of AA, ω-3 AA (i.e., the major C20:4 in N2 (WT)) and JuA to the diet of the *fat-3(wa22)* mutant strain showed significant recovery of growth and development defects (Figure 2). Upon supplementation with all eicosatetraenoic acids, the *fat-3(wa22)* mutant reached L4 stage/adulthood faster than the non-supplemented controls (Figure 2A) and the dumpy phenotype was abolished (observation). Additionally, hatching and brood size defects were recovered by JuA, ω-3 AA and AA to comparable degrees (Figure 2B) (*** *p* < 0.0001).

**Figure 3:**
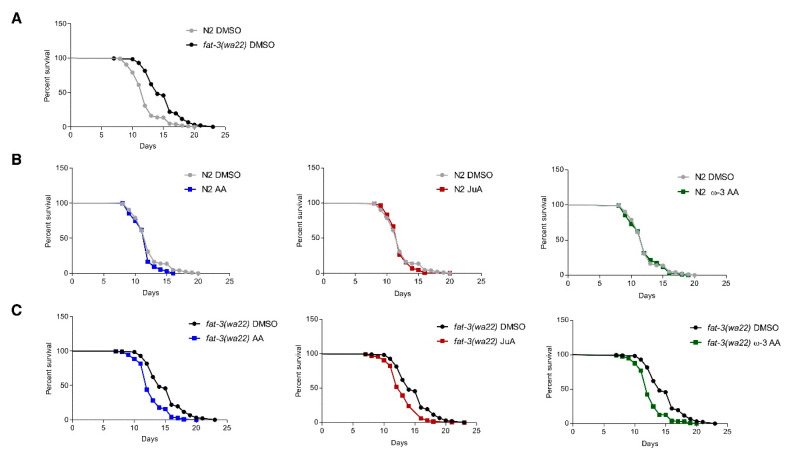
FAs supplementation leads to alteration in lifespans. (**A**) *fat-3(wa22)* mutants are significantly long-lived than N2 (WT) animals (*p* < 0.0001). (**B**) N2 (WT) animal lifespans do not get significantly altered with supplementation of different FAs. (**C**) Lifespans of *fat-3(wa22)* mutants are significantly reduced (with supplementation of different FAs (*p* < 0.0001). All assays were performed at 23 °C with 50 μg/mL FUDR. At least two biological replicate experiments were performed per condition. Statistical analyses were performed in Graphpad Prism 5.0 by using Kaplan–Meier lifespan analysis and *p* values were calculated using the log-rank test. *p* < 0.05 was accepted as statistically significant.

Moreover, we also wanted to access the effect of supplementation of these compounds on the lifespan of *fat-3(wa22*) mutants. Unexpectedly, the mutant strain showed a significantly increased life span in our experimental setup (Figure 3A), contrary to other reports showing that *fat-3(wa22)* animals have a shortened lifespan or no difference in the lifespan when compared to the wild-type [34,35]. Supplementation with AA, ω-3 AA and JuA did not affect the lifespan of N2 (WT) animals (Figure 3B). On the other hand, in the *fat-3(wa22)* mutant strain, all eicosatetraenoic acids likewise reduced the lifespan to the degree observed in the N2 (WT) (Figure 3C), possibly due to an overall rescue of the metabolic deficits and growth alterations, thus leading to a reduced lifespan. These data strongly indicate that AA, ω-3 AA and JuA exert similar biological effects in *C. elegans.*

### 3.3. Elucidation of JuA Biosynthesis in fat-3(wa22) Mutants

**Figure 4:**
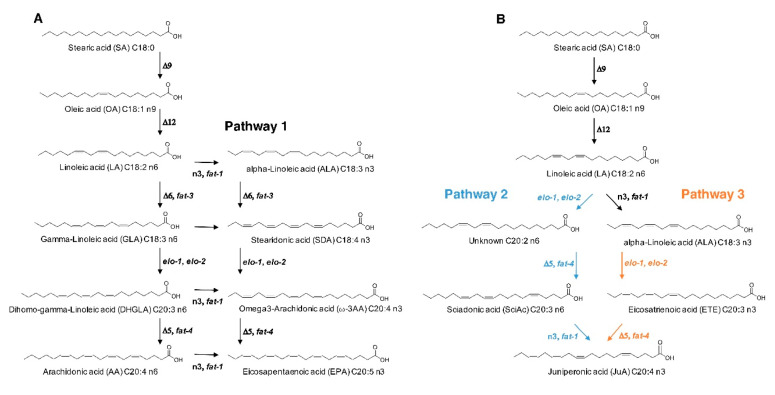
Schematic representation of polyunsaturated fatty acid (PUFA) generation in *C. elegans*. (**A**) Model of generation of PUFAs under normal conditions in N2 (WT) with the intact enzymatic machinery, where LA and ALA act as precursors for generation of PUFAs (AA and EPA). (**B**) Model of novel PUFA synthesis in *fat-3(wa22)* mutants, where ALA acts as the precursor for synthesis of JuA.

The general biosynthetic pathway of AA and ω-3 AA in *C. elegans* is illustrated in Figure 4 (pathway 1). It has previously been reported that the *fat-3(wa22)* mutant contains the C-18 PUFA *α*-linolenic acid (18:3n3) (ALA) that is not present in WT animals [22].

**Figure 5:**
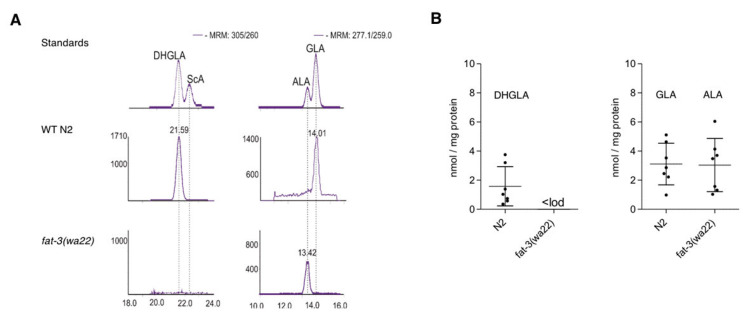
Detection of C20:3 and C18:3. (**A**) Detection of DHGLA, GLA and ALA in N2 (WT) and *fat-3*(*wa22*) animals; no ScA was detected in the *fat-3*(*wa22*) animals. (**B**) Corresponding quantification by LC-MS/MS of the mentioned C20:3 and C18:3. Data represent values from independent experiments. Scatter plots show mean ± SD.

We could reproduce this finding and also show that DHGLA and GLA are missing in the *fat-3(wa22)* mutant (Figure 5). We hypothesized that ALA might act as a precursor for the generation of JuA via one elongation step to generate the intermediate eicosatrienoic acid (C20:3n3) previously identified in the *fat-3(wa22)* mutants [22], which could be further desaturated to form JuA (Figure 4, pathway 3). We ruled out pathway 2 (Figure 4) via linoleic acid (LA) elongation and subsequent desaturation leading to ScA as an intermediate because of the absence of ScA (Figure 5). *C. elegans* has two elongases that mediate the generation of PUFAs, ELO-1 and ELO-2 [22,36,37].

**Figure 6:**
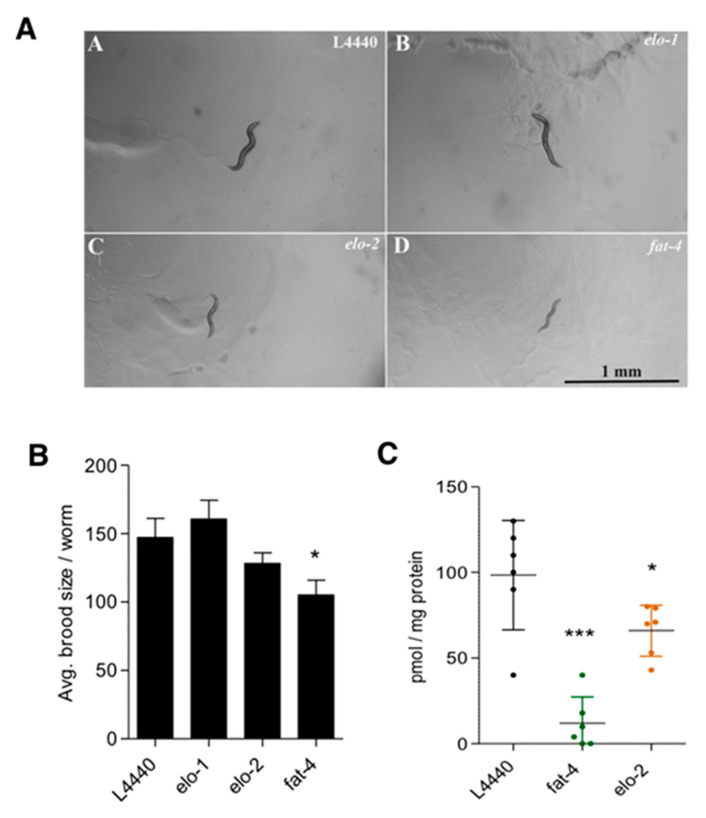
ELO-2 and FAT-4 required for synthesis of JuA. (**A**) shows the effect of RNAi mediated knockdown of elongases and Δ5 desaturase in *fat-3(wa22)* animals. *elo-2* and *fat-4* knockdowns show growth delay after 48 h of hatching at 23 °C as compared *L4440* controls (Scale bar = 100 mm). (**B**) A slight decrease in brood size was observed with the *elo-2* knockdown. A significant reduction in brood size was observed with the *fat-4* know-down (* *p* < 0.01). Data show mean values ± SD. (**C**) Significant reduction in the levels of JuA after the knockdown of *fat-4* (*** *p* < 0.0001) and *elo-2* (* *p* < 0.01) in *fat-3(wa22)* animals. All brood size experiments were carried out with *n* = 5 animals per condition in three independent trials. Scatter plot shows mean ± SD.

The marked increase of ALA suggested that ELO1/2 might form the C20:3n3, which could then be desaturated by *fat-4* to provide JuA (Figure 4, pathway 3). ELO-2 regulates C18 and C20 PUFA elongation together with the condensing enzyme ELO-1 [22,38]. To block pathway 3 (Figure 4), we carried out RNAi knockdowns of both *elo-1* and *elo-2* and determined both the phenotypic effects and changes in the amounts of JuA in the *fat-3(wa22)* mutants. While we observed a further growth delay and slight reduction in brood size in the *elo-2* knockdown, this was not seen with the *elo-1* knockdown (Figure 6A,B).

**Figure 7:**
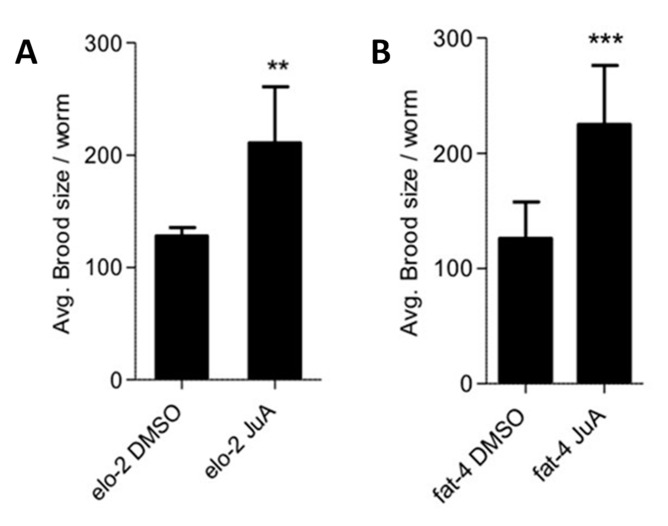
Recovery of brood size of *fat-3(wa22) C. elegans* RNAi knockdowns. Brood size of *fat-3(wa22) C. elegans* where RNAi knockdowns of *elo-2* and *fat-4* were performed showing a significant improvement after supplementation of JuA. (**A**) Recovery in brood size in *elo-2* (** *p* < 0.001) *C. elegans.* (**B**) Recovery in brood size in *fat-4* (*** *p* < 0.0001). All brood size experiments were carried out with *n* = 5 animals per condition in three independent trials. Data show mean values ± SD.

In agreement, there was a significant (*p* < 0.01) albeit moderate reduction in the levels of JuA in the *elo-2* knockdown (Figure 6C), suggesting that this enzyme is involved in elongating ALA to C20:3n3. Next, we knocked down *fat-4* to see whether we observe the expected changes in the levels of JuA. As shown in Figure 6C, the levels of JuA were significantly reduced and almost abolished in this strain (*p* < 0.0001). Importantly, the *fat-4(wa22)* knockdown not only led to a delay in growth but also a significant (*p* < 0.01) reduction in the brood size (Figure 6A,B). Supplementation of JuA could reverse the additional detrimental effects observed in the *fat-4* and *elo-2* knockdowns (Figure 7). These data indicate that the Δ5 desaturase (*fat-4*) is crucial and rate-limiting for the generation of JuA in the *fat-3(wa22)* mutants.

### 3.4. Detection of Different ω-3 Endocannabinoids in C. elegans WT and fat-3(wa22) Mutant Strain

**Figure 8:**
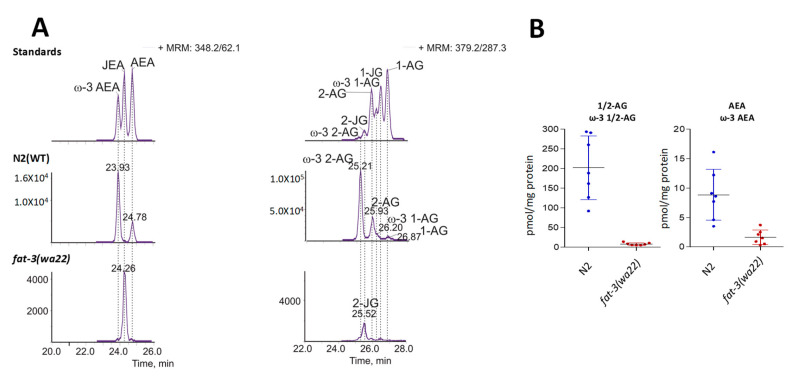
Detection of new EC-like molecules in N2 (WT) and *fat-3(wa22)* animals. (**A**) LC-MS/MS chromatograms of AA, ω-3 AA and JuA derived EC-like molecules in simultaneously bred N2 (WT) animals and *fat-3(wa22)* mutants. (**B**) Quantification of the glycerol derived 1/2-AG, ω-3 1/2-AG and 1/2-JG of the ethanolamine-derived AEA, ω-3 AEA and JEA in N2 (WT) and *fat-3(wa22) C. elegans* by LC-MS/MS. Scatter plots show mean ± SD.

Given our interest in the endocannabinoid system (ECS) and the recent report on JuA derived EC-like molecules in gymnosperms [6], we next assessed the presence of ECs in the *fat-3(wa22)* mutant strain. While the classical ECs have already been reported in N2 (WT) *C. elegans* (i.e., 1/2-AG and AEA), we identified for the first time the ECs derived from ω-3 AA, namely ω-3 1/2-AG and ω-3 AEA (Figure 8A). Because in the N2 (WT) strain ω-3 AA is more abundant than AA (Figure 1), ω-3 1/2-AG and ω-3 AEA could be the major ECs in *C. elegans*, unlike in mammals. To unambiguously confirm the presence of ω-3 1/2-AG and ω-3 AEA in N2 (WT) *C. elegans*, we synthesized these lipids and used them as standards. The presence of ω-3 1/2-AG and ω-3 AEA was observed using our recently published LC-MS/MS method [39] with the appearance of additional peaks at retention times close to that expected for 1/2-AG and AEA (i.e., overlapping peaks). We complemented the analytical LC-MS/MS method to also quantify the JuA-derived ECs (Figure 8B).

**Figure 9:**
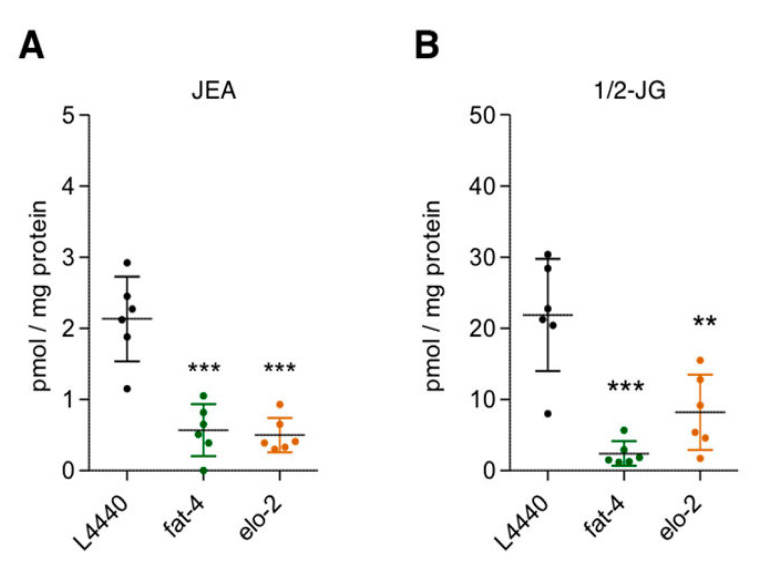
Reduction in the levels of EC like molecules in *fat-3*(*wa22*) after knockdown of *fat-4* and *elo-2.* LC-MS/MS quantification shows that significant reduction in the levels of JEA and 1/2 JG occurs with knockdowns of *fat-4* and *elo-2.* (**A**) Reduction of JEA levels as compared L4440 controls when compared to knockdowns *elo-2* (*** *p* < 0.0001) and *fat-4* (*** *p* < 0.0001). (**B**) Reduction of 1/2 JG levels as compared *L4440* controls when compared to knockdowns *elo-2* (** *p* < 0.001) and *fat-4* (*** *p* < 0.0001). Scatter plots show mean ± SD.

The analysis of the *fat-3(wa22)* mutants revealed the presence of the JuA derived ECs *N*-juniperoyl ethanolamine (JEA) and 1/2 juniperoyl glycerol (1/2-JG) (Figure 8). Matching the overall lower levels of JuA in the *fat-3(wa22)* mutant strain compared to AA in wild type animals, the levels of JuA derived ECs were significantly lower (Figure 8B). There was also a significant decrease in the levels of JEA and 1/2-JG in the *elo-2* and *fat-4* knockdowns of the *fat-3(wa22)* animals, confirming that JuA acts as the biosynthetic precursor of these lipids (Figure 9). Irrespective of the eicosatetraenoic acid present, the corresponding *N*-acylethanolamine and glycerol ester type ECs were generated.

### 3.5. Characterization of AA, ω-3 AA and JuA Aerived Endocannabinoids on Metabolic Enzymes and NPR-32 and NPR-19 Binding

In mammals, AEA and 1/2-AG are primarily degraded by the serine hydrolases fatty acid amide hydrolase (FAAH) and monoacylglycerol lipase (MAGL) [40], respectively. To investigate whether JEA, ω-3 AEA, 1/2-JG and ω-3 1/2-AG effectively compete with classical tritiated ECs for their hydrolysis, we co-incubated them in pig brain homogenate (see methods section). JEA, 1/2-JG and ω-3 1/2-AG were readily hydrolyzed by FAAH and MAGL, respectively. Noteworthy, ω-3 AEA was not competing as efficiently with [^3^H]-AEA hydrolysis in pig brain (IC_50_ >100 µM) as the other *N*-acylethanolamines. Next, we tested hydrolysis competition in *C. elegans*, which has already been shown to express the AEA hydrolyzing enzyme FAAH-1 [41]. In whole worm homogenates JEA, 1/2-JG and ω-3 1/2-AG competed with AEA and 2-OG hydrolysis, respectively. In this assay setup, they were hydrolyzed with similar efficiency as AEA and 2-AG (data not shown). Interestingly, as observed already in pig brain, ω-3 AEA competed less potently with [^3^H] AEA hydrolysis (IC_50_ = 36 µM), suggesting that it is the least favorable substrate for FAAH in this series.

**Figure 10:**
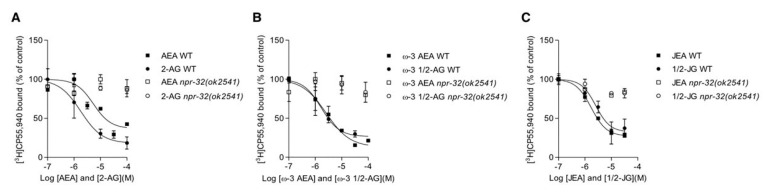
Binding activities of ECs on membrane preparations of N2 (WT) and *npr-32(ok2541)* mutants. WT (full dots) and *npr-32(ok2541)* (empty dots) membranes were incubated with 3 nM [^3^H]CP55,940 in the presence of different concentrations (0.1 to 100 µM) of (**A**) AEA and 2-AG (**B**) ω-3 AEA and ω-3 1/2-AG and (**C**) JEA and 1/2-JG. Data show displacement curves with mean values ± SD of at least three independent experiments each performed in triplicate.

Recently, the GPCRs NPR-19 and NPR-32 were shown to be AEA receptors in *C. elegans* [31], thus providing the first evidence for the existence of EC receptors in *C. elegans*. The study also showed that NPR-19 and NPR-32 affect axon regeneration in the FAAH-1 mutant animals, by sensing AEA [31]. However, no binding or other direct interactions between AEA and these GPCRs were reported. Here, we aimed to establish radioligand binding assays for NPR-19 and NPR-32 using [^3^H]AEA and [^3^H]2-AG. We also employed [^3^H]CP55,940, which is a potent nonspecific CB1/CB2 agonist and GPR55 partial agonist/antagonist [42,43]. While it was not possible to generate a saturation binding curve with the ECs, even when applying PMSF to avoid hydrolysis, a saturation binding curve could be obtained with the ligand [^3^H]CP55,940 with NPR-32 receptors. The calculated B_max_ was 706 ± 120 nM with an apparent *K*_d_ of 2.5 ± 1.8 nM. Despite the relatively high degree of unspecific binding, a reproducible specific dissociation constant could be derived. As shown in Figure 10, ECs were able to compete and displace the specific binding of [^3^H]CP55,940 (30–35%) in N2 (WT) animals. In the *npr-32(ok2541)* deletion mutant, the specific binding was absent (Figure 10), confirming the specific interaction of CP55,940 with NPR-32. On the other hand, by normalizing against NPR-19, no saturation binding curve could be achieved (data not shown). Our data suggest that CP55,940 can be used as a ligand to study binding interactions with NPR-32 in *C. elegans* and exhibits an overlapping binding site with ECs. The classical ECs present in WT animals showed binding affinities to this GPCR with apparent *K_i_* values in the nanomolar to micromolar range. AEA showed a *K*_i_ = 1.9 (95% CI = 0.3–6.2) µM and 1/2-AG *K*_i_ = 450 (95% CI = 377–561). Likewise, the ω-3 eicosatetraenoic acid-derived ECs exhibited significant NPR-32 binding interactions with JEA *K_i_* = 740 (95% CI = 550–811) nM, 1/2-JG *K*_i_ = 1.1 (95% CI = 0.5–6.7) µM, ω-3 AEA *K_i_* = 1.0 (95% CI = 0.3–14) µM and ω-3 1/2-AG *K_i_* = 700 (95% CI = 663–780), which were abolished in the *npr-32(ok2541)* mutants (Figure 10). The ECs 1/2-AG and ω-3 1/2-AG showed the least variability and *K*_i_ values in the upper nanomolar range, in agreement with the estimated concentration range of these lipids in tissues.

## 4. Discussion

The conditionally essential arachidonic acid (AA) is a major n-6 PUFA [44] and AA and docosahexaenoic acid (DHA) make up ∼20% of FAs in the mammalian brain [45]. A deficiency in AA biosynthesis caused by mutations in the rate limiting enzyme Δ6 desaturase (FADS2) leads to significant immune response impairment in mice [46]. An early study about Δ6 desaturase KO (fads2-/-) mice reported infertility issues but no significant effect on lifespan [11]. This mouse strain also showed several detrimental effects in growth and development including infertility, which could be abolished by supplementation of AA [13]. Human studies have suggested that Δ6 desaturase deficiencies might be associated with cutaneous abnormalities, but could also be related to aging [11,47]. Association studies on nucleotide polymorphisms in the FADS locus in humans show important differences in the capacity of different populations to synthesize LC-PUFAs including AA [48]. Recently, in an auxotrophic Δ6 desaturase deficient (fads2 -/-) mouse lacking LC-PUFA synthesis, an impact on the precursor pool of ECs through transformation of eicosa-all cis-5,11,14-trienoic acid into two novel ECs, 20:35,11,14-ethanolamide and 2–20:35,11,14-glycerol was reported [49], thus indicating PUFA plasticity in mammals.

Given the complexity of FA diet-controlled mammalian experiments, in this study we explored the effect of compensating biochemical mechanisms that take place in the *C. elegans fat-3(wa22)* mutant deficient in AA. *C. elegans* has emerged as an attractive and versatile model organism to study ω-3 and ω-6 FA biosynthesis and metabolism [22,37]. The ease of genetic manipulation allows the fast generation of mutants characterized by altered FAs synthesis. This can alter growth and development patterns associated with the lack of these fundamental FAs, similar to humans [50,51]. In *C. elegans*, exogenous supplementation of key FAs can easily restore biosynthetic defects [36].

The absence of a Δ6 desaturase activity implies that the generation of AA and ω-3 AA is prevented, as LA cannot be converted into GLA and ALA into stearidonic acid (SDA), respectively (see Figure 4). The *fat-3(wa22)* mutant cannot produce classical C20 PUFAs, including AA, DGLA, ω-3 AA and EPA. We found that the *fat-3(wa22)* mutant strain can still generate an ω-3 eicosatetraenoic acid that we identified to be juniperonic acid (JuA). JuA was identified for the first time in oils from seeds of gymnosperms like *Juniperus communis* [52]. Recently, while studying the distribution of AA in the plant kingdom, we observed the presence of JuA and associated *N*-acylethanolamine JEA and glycerol ester 1/2-JG in the leaves of 14 of the 16 gymnosperms and *Equisetum trachyodon* (monilophyte) and in two species of *Selaginella* (lycophytes) [6]. To date, JuA has not been detected in Animalia, with the exception of tissues or organs from rats that have been supplemented with JuA-rich biota seed oils [53]. In this study, the altered growth and brood size phenotypes of the *fat-3(wa22)* mutants were recovered when animals were fed with excess of AA or ω-3 AA and partially also with JuA supplementation. Additionally, we observed alterations in the lifespan of the *fat-3(wa22)* mutants. After supplementation of these FAs, the increased lifespan observed in these mutant animals were reduced significantly and the lifespans were normalized, resembling N2 (WT) lifespans, indicating restoration of this important phenotype. Although the lifespan extension observed in the *fat-3(wa22)* mutants is not consistent with what was reported previously, as some studies have shown that these mutants either show no change or reduced lifespans as compared to the N2 (WT) animals [34,35]. This could be explained by one of the key differences between the previous methods and ours. We used FUdR to inhibit the progeny. FUdR supplementation has been known to cause a lifespan extension in a dose dependent manner in N2 (WT) animals and also in some short-lived mutants with altered metabolic patterns [54,55,56,57]. This discrepancy is noteworthy and needs to be explored in future studies. Nevertheless, our data strongly indicates that JuA is generated de novo in the *C. elegans fat-3(wa22)* mutants as a compensatory mechanism to biochemically counterbalance for the general lack of C20s, including ω-3 AA and AA. One study has shown mutants lacking the capability to synthesize C20 PUFAs EPA and AA have overall defects in touch sensation [52]. It was also shown that supplementation of PUFAs including AA in these mutants restores the touch sensation deficit, indicating the crucial role played by these PUFAs in mechanosensation [58]. Touch sensation regulation by C20 PUFA-containing phospholipids may be conserved in mammals because high dietary intake of PUFAs is known to improve nerve conduction in aged individuals [59]. As an additional consequence of the lack of C20:4 in the *fat-3(wa22)* mutants, a delay in growth and a reduction in the brood size was observed (Figure 6A,B). Supplementation with JuA rescued the detrimental effects suggesting the importance of JuA for the viability of these nematodes. The addition of JuA to mammalian cells suppressed MAPK signaling-mediated pro-inflammatory mediator production in murine macrophages and significantly attenuated inflammation in a mouse ear edema model [60]. Moreover, when 3T3 cells are cultured with JuA, cellular ALA accumulates in a concentration-dependent manner associated to JuA concentration, which hints towards the idea that ALA and JuA are interrelated [61].

A possible correlation between ALA and JuA is shown in Figure 4 (pathway 2 or 3), where we propose a feasible biosynthetic pathway for JuA. We assumed that elongase’s ELO-1 and ELO-2 are the enzymes involved in the elongation of the unsaturated chain, which are orthologs of human ELOV3 (ELOVL fatty acid elongase 3) and ELOVL6 (ELOVL fatty acid elongase 6). It has been shown previously that genetic deletion of these enzymes blocks the synthesis of 20-carbon PUFA [38,62]. We carried out RNAi knockdowns of both *elo-1* and *elo-2* in the *fat-3(wa22)* mutants, monitoring the phenotypic changes and the changes in JuA levels and found that *elo-2* knockdown led to an additional reduction in brood size. It should be noted that the *elo-2* knockdown may not necessarily be related to a JuA deficiency, because *C. elegans elo-2* KO have already been associated with many developmental defects that are not observed in the *elo-1* knockouts [38]. Our LC-MS/MS data indicate that ELO-2 is one of the key enzymes involved in ALA elongation to generate the C 20:3 PUFA, which is further transformed to JuA. In the Δ5 desaturase (*fat-4*) knockdown of *fat-3(wa22)* mutants, JuA levels were almost abolished. This is in line with a previous study where worm Δ5 desaturase was expressed in yeast, which when supplied with n-3 fatty acid (eicosapentanoic acid (EPA) as unconventional substrate, was able to generate uncommon, non-methylene interrupted fatty acids (i.e., possibly JuA) [63].

AA originating from different metabolic hubs gives rise to the generation of ECs [64,65]. Given the crucial role played by ECs in various biological functions in animals, including growth and development [66], we investigated if other eicosatetraenoic acids could potentially act as precursors for ECs. EC-like molecules derived from ω-3 AA and JuA were identified here for the first time (Figure 8) in the N2 (WT) and the *fat-3(wa22)* mutant strain, respectively. This confirms the biosynthetic plasticity previously observed in lower plants [6]. Our previous findings on AA, JuA and their ECs and EC-like derived metabolites in the plant kingdom shows a distribution of AA, AEA and 1/2-AG among algae and lower plants (bryophytes and monilophytes) that inversely correlated with JuA, JEA and 1/2-JG present in gymnosperms, lycophytes and a few lichens [6]. It is known that in *C. elegans,* FAAH-1 is able to degrade *N*-acylethanolamine (NAE) type signaling lipids, including AEA. Recently, it was reported that FAAH-4 is a functional homologue of mammalian MAGL, the enzyme responsible of 2-AG hydrolysis [67]. The synthetized EC-like molecules were tested on the hydrolyzing enzymes using pig brain homogenate and whole worm homogenate as enzyme sources. When compared to the known ECs, JEA, 1/2-JG and ω-3 1/2-AG show a similar efficiency in competing for hydrolyzing enzymes. Interestingly, performing the abovementioned assay using a synthetically prepared ω-3 AEA as a substrate shows poor hydrolysis competition with AEA and an estimated IC_50_ >100 µM. This occurred in both pig brain and worm homogenate, revealing comparability between the two systems. In light of the fact that only nanomolar ω-3 AEA levels are found in N2 wild type animals, this lipid may undergo a different metabolism from AA. Further studies are necessary to see if these lipids can also act as precursors for the generation of other novel eicosanoids, which might exert similar biological functions. Classical ECs such as AEA and 2-AG are generated due the actions of enzymes such as *N-*acylphosphatidylethanolamine-hydrolyzing phospholipase D (NAPE-PLD) and diacylglycerol lipase (DAGL). Similarly, *C. elegans* has a homolog of NAPE-PLD that is also necessary for the generation of ECs [65,68]. Additional studies are needed to determine the enzymes responsible for the biosynthesis of the novel ECs.

A recent report suggested that the GPCRs NPR-32 and NPR-19 act as AEA receptors in *C. elegans* [31], providing the first evidence for the existence of EC receptors in *C. elegans*. Hence, we wanted to test the binding efficiency of the conventional ECs and EC like molecules on NPR-32 and NPR-19. We observed that CP55,940, a potent nonselective CB1 and CB2 receptor agonist, was displaced by classic ECs and EC-like molecules derived from ω-3 AA and JuA. Despite a relatively high non-specific binding, a 30–35% of specific displacement was observed in N2 (WT) animals. We were thus able to calculate the *K*_i_ values for the classical ECs (AEA *K_i_* = 1.9 µM, 1/2-AG *K_i_* = 450 nM) and also for the ω-3 eicosatetraenoic acid derived ECs (JEA *K_i_* = 740 nM, 1/2-JG *K_i_* = 1.1 µM, ω-3 AEA *K_i_* = 1.0 µM, ω-3 1/2-AG *K_i_* = 700 nM).

This displacement was not observed in the *npr-32(ok254)* mutants, suggesting that NPR-32 represents a common binding site for ECs and CP55,940. NPR-32 is related to the mammalian GPR18 and GPR55 receptors and is a predicted ortholog of human CCR2 (C-C motif chemokine receptor 2) [31,62]. Studies have also shown that GPR18 and GPR55 are activated by cannabinoids [69,70]. Moreover, AEA had suppressive effects on axon regeneration after injury, which is known to be modulated by NPR-32/19 in *C. elegans* [31]. It has also been shown that CCR2 receptors get induced and are co-expressed with CB1 receptors in the thalamus after severe lesions in animal models of spinal cord injury [71]. These findings point to the possibility that these receptors and newly identified EC-like molecules may play a role in nervous system injury and regeneration. Although in our hands CP55,940 did not bind to NPR-19, another important EC receptor in *C. elegans* [72], we cannot exclude that JEA and 2-JG also activate this receptor. Similar to mammals, the presence of ECs has been shown to play a key role in determining the nutrient status, metabolism and aging in *C. elegans* [73,74]. Additionally, our observations also highlight the role of these lipids in growth/development and other phenotypes, which can be linked to metabolic status. As summarized, previous studies have already shown connections between nutrient status, metabolism and various components of the ECs in mammals [75,76,77]. It would thus be interesting to see if these newly identified EC-like molecules and FAs might play a crucial role in altering and modulating the metabolic status by interacting with key nutrient sensing and signaling pathways.

Taken together, our study uncovered that genetic and metabolic plasticity exists in *C. elegans* in order to bypass mutations that alter the synthesis of AA. Adequate analytical methods able to recognize all stereoisomers of FAs (e.g., C20 PUFAs) are crucial to study the plasticity of eicosatetraenoic acids and underlying biochemical relationships. In this study the use of an optimized LC-MS/MS method enabled the differentiation between ω-3 AA, AA and JuA, respectively. By utilizing simple model organisms such as *C. elegans* to systematically dissect fundamental biological processes, we provide evidence of the plasticity of lipid biology mediated by Δ6 desaturase deficiency. Whether the alternative ALA and JuA eicosatetraenoic pathways are present in mammals with Δ6 desaturase deficiency will be the subject of a follow-up study. Felines lack the biosynthetic machinery to generate AA [8,9], which makes them obligatory carnivores. Δ6 desaturase deficiency in humans has been associated to metabolism, aging, skin abnormalities and neuropsychiatric disorders [14,15,78]. Because dietary AA intake is restricted to animal food products (dietary plants do not contain AA), we postulate that JuA may be generated in individuals that have a vegan diet and are suffering from a Δ6 desaturase deficiency. If the biosynthesis of JuA would be identical to what is observed in worms (Figure 4), there could also be dietary implications, such as the necessary intake of ALA from plant oils.

## Figures and Tables

**Table 1:** Detection of fatty acids (FAs) relevant to this study in wild type N2 (WT) and *fat-3(wa22)* animals.

Fatty Acids	WT	*fat-3 (wa22)*
Arachidonic acid (AA)	+	-
ω-3 arachidonic acid (ω-3 AA)	+	-
Juniperonic acid (JuA)	-	+
α-linoleic acid (ALA)	-	+
γ-linoleic acid (GLA)	+	-
Sciadonic acid (ScA)	-	-
dihomo-γ-linoleic acid (DHGLA)	+	-

## Supplementary Materials

The following are available online at https://www.mdpi.com/2073-4409/9/9/2127/s1, Figure S1: Synthesis of -3AEA (2), Figure S2: Synthesis of ω-3 1-AG (4) and ω-3 2-AG (5). The authors confirm that the data supporting the findings of this study are available within the article [and/or] its Supplementary Materials.

## Abbreviations

1/2-AG	1/2-arachidonoylglycerol
1/2-JG	1/2-juniperoylglycerol
2-OG	2-Mono-oleoyl-rac-glycerol
AA	Arachidonic acid
ACN	Acetonitrile
AEA	N-arachidonoyl ethanolamine
ALA	α-linoleic acid
CCR2	C-C motif chemokine receptor 2
CGC	Caenorhabditis Genetics Center
CHCl_3_	Chloroform
CH_3_OH	Methanol
DAGL	Diacylglycerol lipase
DHGLA	Dihomo-gamma-linolenic acid
ECs	Endocannabinoids
ECS	Endocannabinoid system
ELO 1/2	Fatty acid eongases in C. elegans
EPA	Eicosapentanenoic acid
FAs	Fatty acids
FAAH	Fatty acid amide hydrolase
FADS2	Fatty acid desaturase 2, gene of Δ6 desaturase
FUDR	5′-fluorodeoxyuridine
GLA	Gamma linoleic acid
GPCR	G protein coupled receptor
HCl	Hydrogen chloride
IPTG	Isopropyl-β-D-thiogalactopyranoside
JEA	N-juniperoyl ethanolamine
JuA	Juniperonic acid
LA	Linoleic acid
LC	Liquid chromatography
MAGL	Monoacylglycerol lipase
MgCl_2_	Magnesium chloride
NAE	N-acylethanolamine
NAPE-PLD	N-acylphosphatidylethanolamine-hydrolyzing phospholipase D
NGM	Nematode growth media
NPR-19	Cannabinoid G-protein coupled receptor in C. elegans
NPR-32	Cannabinoid G-protein coupled receptor in C. elegans
PUFAs	Polyunsaturated fatty acids
ScA	Sciadonic acid
ω -3 AEA	Omega-3 N-arachidonoyl ethanolamine
ω -3 1/2-AG	Omega-3 1/2-arachidonoyl glycerol
ω-3 AA	Omega-3 arachidonic acid
