# Supplementary material for: Juniperonic Acid Biosynthesis is Essential in Caenorhabditis elegans Lacking Δ6 Desaturase (fat-3) and Generates New ω-3 Endocannabinoids
Source: Cells. 2020 Sep 19;9(9):2127. doi: 10.3390/cells9092127 (PMC7564282; doi:10.3390/cells9092127)
Supplement: Supplementary file 1 [file cells-09-02127-s001.pdf]

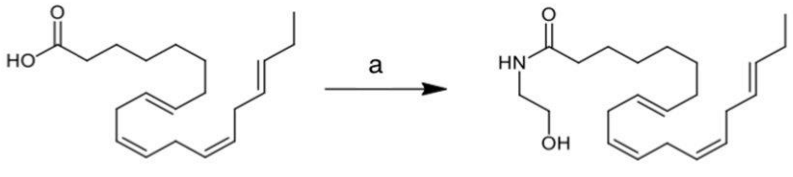

1 2

Figure S1. Synthesis of ⅂-3AEA (2). Reagent and conditions: (a) (1) $(COCl)_2$, DMF, CH_2Cl_2, 0° to r.t., 4h; (2) Ethanolamine, CH_2Cl_2, r.t., overnight.

^{1}H-NMR (300MHz, $CDCl_3$) δ (ppm): 5.40-5.25 (m, 8H), 3,69 (t, 2H, *J*=4.95 Hz), 3.38 (t, 2H, *J*=5.1 Hz), 2.78 (m, 6H), 2.16 (t, 2H, *J*= 8.5 Hz), 2.08-1.98 (m, 4H), 1.64 (t, 2H, *J*=7.5 Hz), 1.34-1.28 (m, 6H), 0.93 (t, 3H, *J*= 7.5).

^{13}C-NMR (200 MHz, $CDCl_3$) δ (ppm): 174.58, 131.88, 130.19, 128.50, 128.42, 128.00, 127.96, 127.76, 127.03, 62.65, 42.53, 36.64, 29.69, 29.44, 29.17, 28.96, 27.16, 25.64, 25.55, 20.61, 14.46.

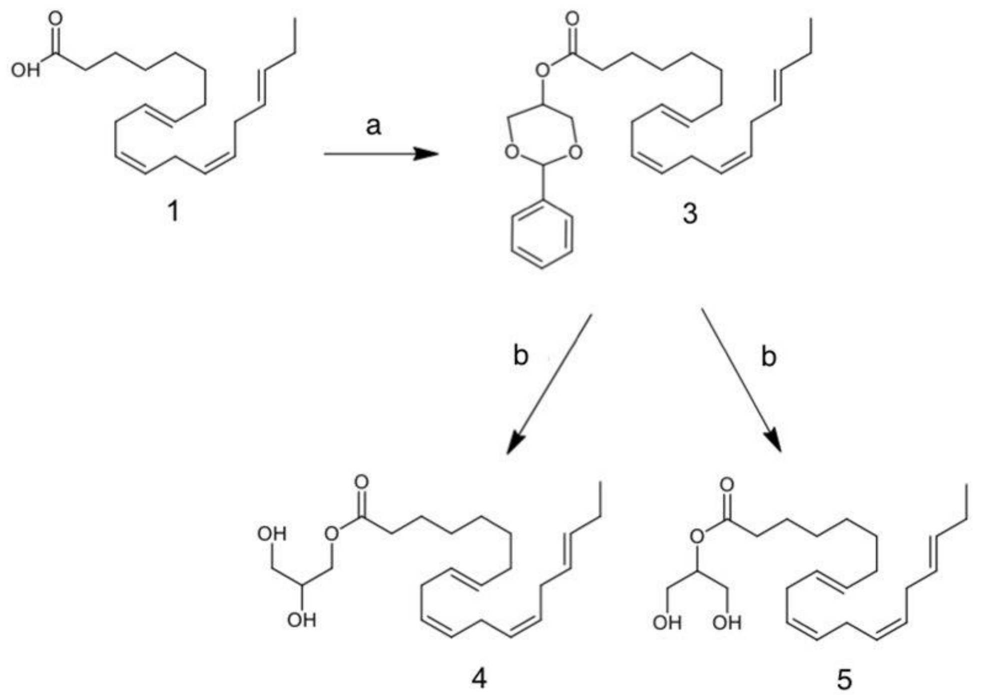

Figure S2. Synthesis of ω-3 1-AG (4) and ω-3 2-AG (5). Reagent and conditions: (a) 1) $(COCl)_2$, DMF, CH_2Cl_2, 0° to r.t., 4h; 2) cis-1,3-O-Benzylideneglycerol, CH_2Cl_2, r.t., 24h, dark; (b) B-chlorocatecholborane, CH_2Cl_2, r.t., 2h, dark.

^{1}H-NMR (300MHz, $CDCl_3$) δ (ppm): 5.45-5.28 (m, 8H), 4.23-4.14 (m, 2H), 3.96-3.83 (m, 2H), 3.69 (dd, 1H, *J*=3.3, 11.4 Hz), 3.60 (dd, 1H, *J*=4.3, 11.4 Hz), 2.83 (m, 6H), 2.35 (t, 2H, *J*= 5.7 Hz), 2.11-2.03 (m, 4H), 1.63 (t, 2H, *J*=5.4 Hz), 1.33-1.25 (m, 6H), 0.97 (t, 3H, *J*=5.7).

^{13}C-NMR (200 MHz, $CDCl_3$) δ (ppm): 174.58, 132.03, 130.15, 128.51, 128.42, 128.02, 127.96, 127.79, 127.03, 70.34, 65.25, 63.37, 34.11, 29.73, 29.44, 29.06, 28.86, 27.13, 25.69, 25.53, 24.83, 20.55, 14.24.
